# *In Vivo* Immunostaining of Hemocyte Compartments in *Drosophila* for Live Imaging

**DOI:** 10.1371/journal.pone.0098191

**Published:** 2014-06-03

**Authors:** Gábor Csordás, Gergely I. B. Varga, Viktor Honti, Ferenc Jankovics, Éva Kurucz, István Andó

**Affiliations:** 1 Immunology Unit, Institute of Genetics, Biological Research Centre of the Hungarian Academy of Sciences, Szeged, Hungary; 2 Developmental Genetics Unit, Institute of Genetics, Biological Research Centre of the Hungarian Academy of Sciences, Szeged, Hungary; Uppsala University, Sweden

## Abstract

In recent years, *Drosophila melanogaster* has become an attractive model organism in which to study the structure and development of the cellular immune components. The emergence of immunological markers greatly accelerated the identification of the immune cells (hemocytes), while the creation of genetic reporter constructs allowed unique insight into the structural organization of hematopoietic tissues. However, investigation of the hemocyte compartments by the means of immunological markers requires dissection and fixation, which regularly disrupt the delicate structure and hamper the microanatomical characterization. Moreover, the investigation of transgenic reporters alone can be misleading as their expression often differs from the native expression pattern of their respective genes. We describe here a method that combines the reporter constructs and the immunological tools in live imaging, thereby allowing use of the array of available immunological markers while retaining the structural integrity of the hematopoietic compartments. The procedure allows the reversible immobilization of *Drosophila* larvae for high-resolution confocal imaging and the time-lapse video analysis of *in vivo* reporters. When combined with our antibody injection-based *in situ* immunostaining assay, the resulting double labeling of the hemocyte compartments can provide new information on the microanatomy and functional properties of the hematopoietic tissues in an intact state. Although this method was developed to study the immune system of *Drosophila melanogaster*, we anticipate that such a combination of genetic and immunological markers could become a versatile technique for *in vivo* studies in other biological systems too.

## Introduction

Fluorescence-based imaging techniques are widely used in studies relating to the development of the hematopoietic system, and in tumor biology and immunity in general. Due to the similarities of the innate immune responses in vertebrates and in insects, its powerful genetic system has led to *Drosophila melanogaster* becoming a key model organism of innate immunity [1]–[3].

The hemocytes in *Drosophila* fall into three categories: plasmatocytes, crystal cells and lamellocytes. Plasmatocytes are small round cells that clear microbes by phagocytosis [4], and produce antimicrobial peptides and extracellular matrix components [5], [6]. Crystal cells contain high amounts of the prophenol oxidases required for melanization [7]. Lamellocytes, the large, flat, key effector cells of the encapsulation reaction, appear after immune induction by the eggs of parasitic wasps or in response to tumors [8], [9].

The hemocytes of the *Drosophila* larva populate three hematopoietic compartments. In the *circulation*, the cells move freely in the body cavity, pumped by the dorsal vessel. The *lymph gland*, a compact, multilobular hematopoietic organ attached to the anterior portion of the dorsal vessel [10], [11], comprises plasmatocytes and crystal cells in its cortical zone, and progenitor cells in its medullary zone [12]–[14]. In the *sessile hematopoietic tissue*, the hemocytes adhere to the subepithelial layer of the body cavity, forming a striped pattern along the longitudinal axis of the larva [15]–[17].

The identification of *Drosophila* hemocytes initially relied on morphological criteria [18]. However, the advent of hemocyte subset-specific molecular markers allowed a clear definition of morphologically and functionally distinct effector cell types [19]–[21]. All larval hemocytes express a highly glycosylated transmembrane protein, Hemese, a member of the sialophorin protein family [19]. Plasmatocytes express the transmembrane protein NimrodC1, identified as a bacterium-binding phagocytosis receptor [20], [22]. Although these markers have become essential tools for the characterization of hemocytes and hematopoietic tissues in *ex vivo* samples, the delicate structure of the immobile hemocyte compartments, and especially that of the sessile tissue, is disrupted, which severely hinders their comprehensive structural analysis.

The construction of *in vivo* reporters and hemocyte-specific GAL4 lines in recent years [15], [23]–[26] allowed a detailed anatomical and functional characterization of the hematopoietic compartments, and in particular the lymph gland [12], [13] and the sessile hematopoietic tissue [16], [17], [27], [28].

We set out to complement the *in vivo* reporters with immunological markers in live larvae with a view to studying the composition and structure of the hematopoietic tissues in an undisturbed state. This requires a simple and effective immobilization of the larva for the duration of the microscopic analysis. This was earlier achieved by dissection [29], [30], by the use of chloroform [31], through the administration of CO_2_ or isofluorane to the larva [32]–[34], or by placing the specimen in a specially prepared microfluidic chamber and applying vacuum [35]. Isofluorane was found to be very effective, but it arrests the pulsation of the dorsal vessel [32], [33] thereby interfering with the circulation of the hemolymph and the mobile hemocytes. We present here an effective and simple method with which to paralyze the larva for an extended period by the use of an acetylcholinesterase inhibitor. This method of immobilization, combined with the genetic and immunological tools mentioned above, allows the *in situ* examination and analysis of the hematopoietic compartments with a so far unprecedented resolution.

## *Drosophila* Stocks and Materials

### *Drosophila* stocks

Flies were kept on cornmeal-yeast food at 25°C. *R3-Hml>GFP* (*R3-w^1118^; Hml.delta.GAL4, UAS-2xEGFP*) [36] was used to visualize plasmatocytes, and *atilla^minos^* (*Mi{ET1}atilla^MB05359^*) [37] to detect lamellocytes; *atilla^minos^; l(3)mbn^1^* (*Mi{ET1}atilla^MB05359^; l(3)mbn^1^/TM6, Tb*) [37] is a tumor suppressor mutant with proliferating tumorous hemocytes and GFP-marked lamellocytes in the hematopoietic compartments. The *Hml>GFP* (*w^1118^; Hml.delta.GAL4, UAS-2xEGFP*) line was used as a P1 negative control.

### Antibodies

The mouse monoclonal antibody anti-Hemese (1.2) [19] reacts with all hemocytes in the larva, the anti-NimC1 reagent, a mixture of P1a and P1b antibodies, reacts with plasmatocytes [20], and T2/48, a negative control antibody, reacts with the human CD45 molecule [21]. These antibodies were used as tissue culture supernatants, at an immunoglobulin concentration of 15 µg ml^−1^. The secondary antibody was an anti-mouse Alexa-633 conjugate (Invitrogen) goat polyclonal antibody, or an anti-mouse CF-568 conjugate (Sigma-Aldrich) goat polyclonal antibody.

## Methods

### Immobilization of the larvae

Larvae were placed in a 25-µl droplet of *Drosophila* Ringer's solution containing Dichlorvos (Fluka, diluted 1∶1000) for 5 min at 25°C, and then transferred into glass-bottom dishes (Cell E&G). To ensure the stability of the larvae, the coverslip was coated with glue. The glue was dissolved in 80 ml heptane from the surface of a 1-m-long double-sided adhesive tape (3M #415) for 24 h at 25°C, after which the tape was removed. This solution was layered onto the coverslip and dried [38]. To prevent desiccation, larvae were mounted with 10S Voltalef oil (VWR).

### Confocal microscopic analysis

Samples were analyzed by means of a Leica TCS SP5 II confocal microscope. The images were merged stacks of 15 slices, combined in ImageJ (maximum intensity stacking). The frames of Video S1 and Video S2 were stacks of 5 slices, combined and sequenced using ImageJ at 5 frames per second (as in [39]).

### Preparation of the antibodies for injection

Monoclonal antibodies to Hemese (1.2), to NimrodC1 (a mixture of P1a and P1b), and against human CD45 (T2/48) (negative control) were used in the form of hybridoma-culture supernatants. The secondary antibody was added to the respective supernatant in 1∶1000 final dilution. This mixture was incubated for 10 min at 25°C to generate hemocyte-specific immunocomplexes, prior to injection.

### Antibody injection

Third instar larvae were washed in *Drosophila* Ringer's solution, and placed on a dry paper towel. With a sharpened glass capillary, 1 µl of the mixture of the primary and secondary antibodies was injected into the hemocoel of third instar larvae, near the posterior end, between segments A6 and A7.

### Preparation of circulating hemocytes

All procedures described below were performed at room temperature (20°C). Larvae were dissected on 12-spot microscope slides (SM-011, Hendley-Essex) in Shields & Sang medium containing 1-phenyl-2-thiourea (PTU). Hemocytes were left to adhere for 45 min, after which they were fixed in paraformaldehyde (2 per cent, in PBS) for 12 min and washed three times in PBS for 5 min. The samples were then blocked with PBS containing 0.1 per cent bovine serum albumin (PBS-BSA) for 15 min.

### Staining of circulating hemocytes with immunocomplexes

The mixture of the respective hybridoma supernatants (anti-Hemese, anti-NimrodC1 or T2/48) and the secondary antibody (anti-mouse CF-568 conjugate, Sigma-Aldrich) in a ratio of 1000∶1 was prepared and incubated for 5 min. The prepared hemocyte samples were treated with the mixture of antibodies for 1 h, and were washed three times with PBS for 5 min. The nuclei were stained with DAPI (Sigma-Aldrich). The samples were mounted with Fluoromount-G (SouthernBiotech) and investigated with a Zeiss Axioskope 2 MOT fluorescent microscope.

### Staining of circulating hemocytes with sequential indirect immunofluorescence

The prepared samples were treated with the hybridoma supernatants for 1 h, followed by three washes in PBS for 5 min. The secondary antibody (anti-Mouse CF-568, Sigma-Aldrich) was applied to the sample in a dilution of 1∶1000 in PBS-BSA for 45 min. The nuclei were stained with DAPI. The sample was washed three times with PBS, mounted with Fluoromount-G and inspected with a Zeiss Axioskope 2 MOT fluorescent microscope.

## Results

### Immobilization of the *Drosophila* larvae

**Figure 1:**
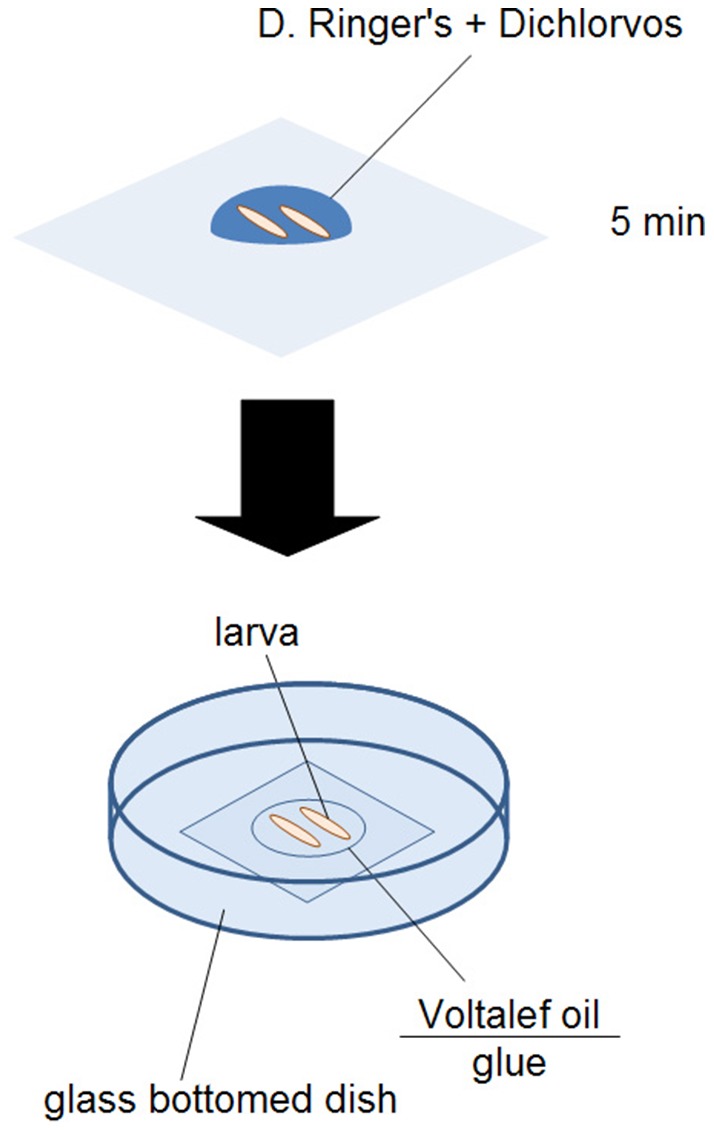
Preparation of larvae for *in vivo* microscopic analysis.

**Figure 2:**
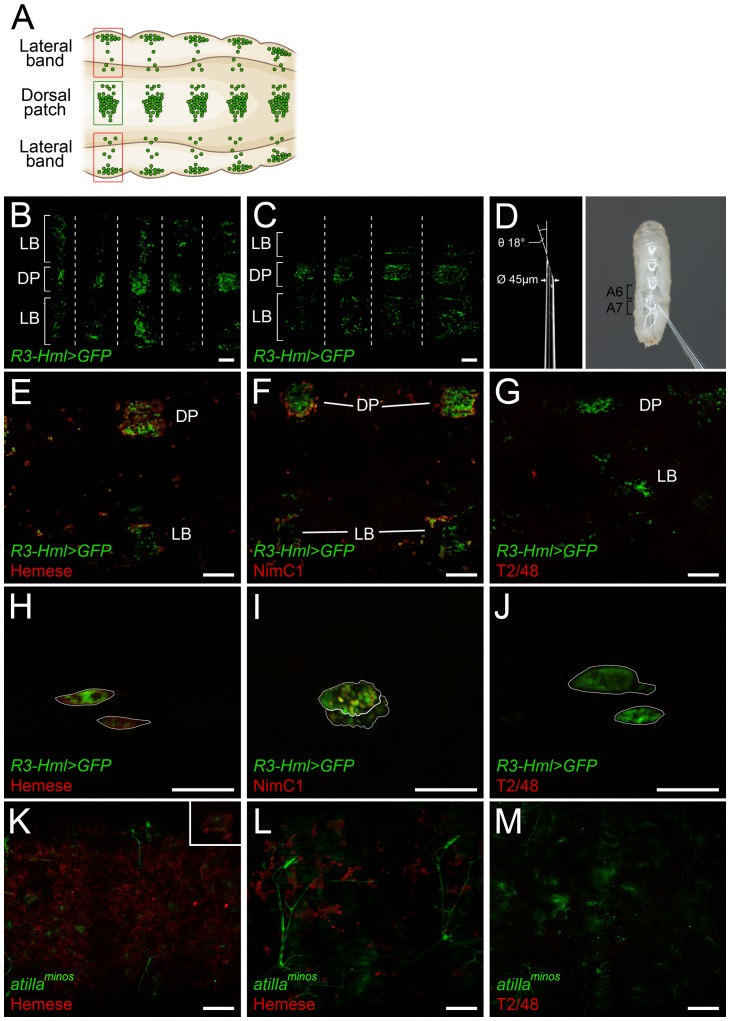
The schematic representation of the larval sessile hematopoietic tissue (A). The sessile tissue of an immobilized *R3-Hml>GFP* larva (B), and a mock-injected *R3-Hml>GFP* larva (C). The dorsal patches are indicated by DP, and the lateral bands are indicated by LB. Segmental borders are marked with dashed lines. The parameters for the sharpened capillaries used for the injection of larvae, and a high-magnification photograph of the injection (D). Staining (red) of the sessile hemocytes (green) in *R3-Hml>GFP* larva with anti-Hemese (E), anti-NimC1 (F) and T2/48 (G) antibodies. The lymph glands (outlined in white) of *R3-Hml>GFP* larvae (green) stained *in situ* with anti-Hemese (H), anti-NimC1 (I) or T2/48 (J) antibodies (red). The sessile hematopoietic tissue of *atilla^minos^; l(3)mbn^1^* (K) and *atilla^minos^* (L) larvae stained for Hemese (red). Negative control staining of *atilla^minos^; l(3)mbn^1^* larva with T2/48 antibody (M). The lamellocytes (green) of *atilla^minos^; l(3)mbn^1^* larva in the hematopoietic tissue stained for Hemese (K, insert), indicated by the arrows. All scale bars indicate 50 µm.

Each larva was physically immobilized in a 25-µl droplet of Dichlorvos (Fluka) solution, diluted in *Drosophila* Ringer's solution. Dichlorvos (2,2-dichlorovinyl dimethyl phosphate), is an acetylcholinesterase inhibitor. In preliminary experiments (not shown), exposure at a dilution of 1∶1000 in *Drosophila* Ringer's solution for 5 min at 25°C paralyzed the larvae for over 1 h. For microscopic analysis, larvae were placed on glue-covered glass-bottom dishes, and mounted with Voltalef 10S oil, as shown in Figure 1. The immobilization of the larvae did not affect the characteristic structure (Figure 2A) and the integrity of the sessile hematopoietic compartment (Figure 2B).

### The hemocyte compartments of the paralyzed larvae

Paralyzed *R3-Hml>GFP* larvae were subjected to confocal microscopic time-lapse analysis (Video S1). Examination of the generated video files revealed that the hematopoietic compartments were intact, the pulsation of the dorsal vessel was not influenced and the circulation of the hemocytes in the dorsal vessel and in the hemocoel was normal. In some cases, minor movements of the larva were noticed (Video S1), but these did not affect the video-analysis.

### *In vivo* immunostaining of hemocytes

With a glass capillary, third instar *R3-Hml>GFP* larvae were injected with the mixture of antibodies to established hemocyte-specific markers [19]–[21] and far-red-fluorescence-labeled secondary antibodies (Figure 2D), and the hematopoietic tissues were analyzed with the aid of a confocal microscope 15 min after the injection. Although the injury and the volume injected had no effect on the structure of the sessile tissue (Figure 2C) or the other compartments (not shown), elongation of the larva was observed (Figure 2C). Mixtures of anti-Hemese or anti-NimC1 antibodies and secondary antibodies stained membranes of hemocytes in the sessile compartment (Figure 2E, F), while the negative control antibody (T2/48) gave no signal (Figure 2G). Analysis of the samples confirmed that the expressions of the *in vivo* marker *R3-Hml>GFP* and the Hemese antigen overlapped. Moreover, the NimC1 marker was expressed in more than 80 per cent of the *R3-Hml>GFP*-positive sessile hemocytes. In the lymph gland, the anti-Hemese antibody (Figure 2H) reacted with the whole surface of the lymph gland, whereas the anti-NimC1 antibody gave a patchy staining pattern in the cortical zone of the primary lobes (Figure 2I). The negative control antibody, T2/48, did not give a signal (Figure 2J). In order to confirm that the specificity of the antibody was not altered in the immunocomplex, circulating hemocytes from *R3-Hml>GFP* larvae were immunostained with sequential indirect immunofluorescent staining and with the mixture of primary and secondary antibodies (Figure S1). Even though the staining of the antibody mixture was weaker (Figure S1B) than the staining of the sequential method (Figure S1A), no difference was observed in the staining pattern of the two procedures. Furthermore, to provide a genetic control, a NimC1 negative *Hml>GFP* sample was also included and was stained against NimC1; no staining was observed in either case (Figure S1).

To generate time-lapse confocal videos from *in situ* immunostained specimens, larvae were injected with a mixture of anti-Hemese antibody and Alexa-633-conjugated secondary antibody, paralyzed and mounted on glass-bottomed dishes. Twenty minute video recordings showed the specific far-red staining of GFP-expressing hemocytes (Video S2). As no changes in signal intensity were detected during the experiment, we concluded that this method is a viable option for the creation of time-lapse series of *in situ* immunostained larvae.

Malignant tissue transformations are known to trigger a cellular immune response, and cause blood cell proliferation and differentiation [40]. Since these events may affect the composition and structure of the hematopoiteic compartments, we tested and validated the *in situ* immunostaining technique in tumorous animals. Third instar *l(3)mbn^1^* homozygous larvae contain approximately 150 times as many circulating hemocytes as in uninduced wild-type controls, with severely swollen and often melanized lymph glands [40]. Since no information was available on the state of the sessile hematopoietic tissue in this tumorous mutant, we investigated the sessile compartment of homozygous *atilla^minos^; l(3)mbn^1^* larvae, which also carry an *in vivo* GFP reporter of the Atilla (L1) lamellocyte-specific marker [37]. The larvae were injected with a mixture of anti-Hemese and the far-red-labeled secondary antibody and the sessile hematopoietic tissue was analyzed with confocal microscopy. The structure of the sessile tissue of the mutant larvae was altered (Figure 2K) as compared with that of the non-tumorous *atilla^minos^* control (Figure 2L). Double-positive lamellocytes, displaying both GFP expression and staining for the pan-hemocyte Hemese marker, were clearly visible in the sessile tissue (Figure 2K, insert).

## Discussion

The localization of cells and the structure of tissues in the organism is generally studied on dissected specimens by light microscopy or a high-resolution confocal microscopic analysis after the use of histochemical or immunofluorescent techniques. These methods have a number of limitations: in consequence of their invasive nature of the preparation and the fixation procedures, the fine structure of the tissues and the cell morphology are often disrupted. Live imaging overcomes most of these limitations. Since the translucent cuticle of the *Drosophila melanogaster* embryo and the larva makes such investigations much easier, it has become an ideal model organism for *in vivo* imaging studies.

Analysis of the hematopoietic compartments and the cell-mediated immunity of *Drosophila* has played an important role in our understanding of the vertebrate hematopoiesis and immune response. Most of our knowledge on *Drosophila* hemocytes was gained through the labeling of different cell types with cell-type-specific antibodies [19]–[21], and the use of transgenic reporter constructs [13], [15]–[17]. *In vivo* labeling has been utilized to visualize hemocyte migration in the embryo [25] and also in genetic screens to identify factors that regulate the structure of the sessile hematopoietic tissue [27].

We have presented here a simple approach through which to amalgamate the strengths of these methods. Immobilization of the larvae renders the experimental subjects accessible for confocal microscopic studies, and thereby allows the high-resolution visualization of hemocytes in their normal environment (*e.g.* in the sessile tissue). As the larvae survive the immobilization, their hematopoietic tissues can be investigated throughout the whole of the larval life. This novel method permits the immunostaining of hemocytes *in situ*, and this has proved to be very useful when no transgenic reporter is available to label a certain hemocyte subset, or when two distinct hemocyte populations are to be labeled. The present study has confirmed that the expressions of the *in vivo* marker *R3-Hml>GFP* and the pan-hemocyte Hemese antigen overlap. Moreover, it has demonstrated that the NimrodC1 markers are present on more than 80 per cent of *R3-Hml>GFP*-expressing sessile hemocytes. These results indicate that the method is suitable for the investigation of overlapping or differential expressions of *in vivo* and immunological markers.

Investigation of tumorous *atilla^minos^; l(3)mbn^1^* larvae revealed that the sessile tissue in these specimens is more massive than in the *atilla^minos^* control larvae, and additionally includes fully differentiated lamellocytes.

The combination of immunostaining with transgenic reporters allows the *in situ* investigation of multilabeled hemocytes, and monitoring of the expression of several independent markers on hemocytes throughout the course of the development or during the immune response. As the technique is easy to perform, such an antibody injection may also facilitate the *in situ* investigation of other arthropod model species in which there are no transgenic reporter constructs, but where immunological markers are available, such as *Manduca sexta* [41], *Bombyx mori* [42] or *Anopheles gambiae* [43].

## Supporting Information

Figure S1 **Immunostaining of circulating hemocytes with the sequential indirect immunofluorescent method (A), and the mixture of antibodies (B).** The staining is shown in red, and the hemocytes are marked by their GFP expression (green), and DAPI nuclear staining (blue). The scale bar indicates 50 µm.
(TIF)

Video S1 **The sessile hematopoietic tissue (green) of an immobilized** ***R3-Hml>GFP*** **larva.** The video was rendered with 5 frames per second, and represents 20 min of recording.
(AVI)

Video S2 **The sessile hematopoietic tissue (green) of an immobilized** ***R3-Hml>GFP*** **larva immunostained** ***in situ*** **with anti-Hemese (red).** The video was rendered with 5 frames per second, and represents 20 min of recording.
(AVI)
