# Supplementary figures and images for: In Vivo Immunostaining of Hemocyte Compartments in Drosophila for Live Imaging
Source: PLoS One. 2014 Jun 3;9(6):e98191. doi: 10.1371/journal.pone.0098191 (PMC4043501; doi:10.1371/journal.pone.0098191)

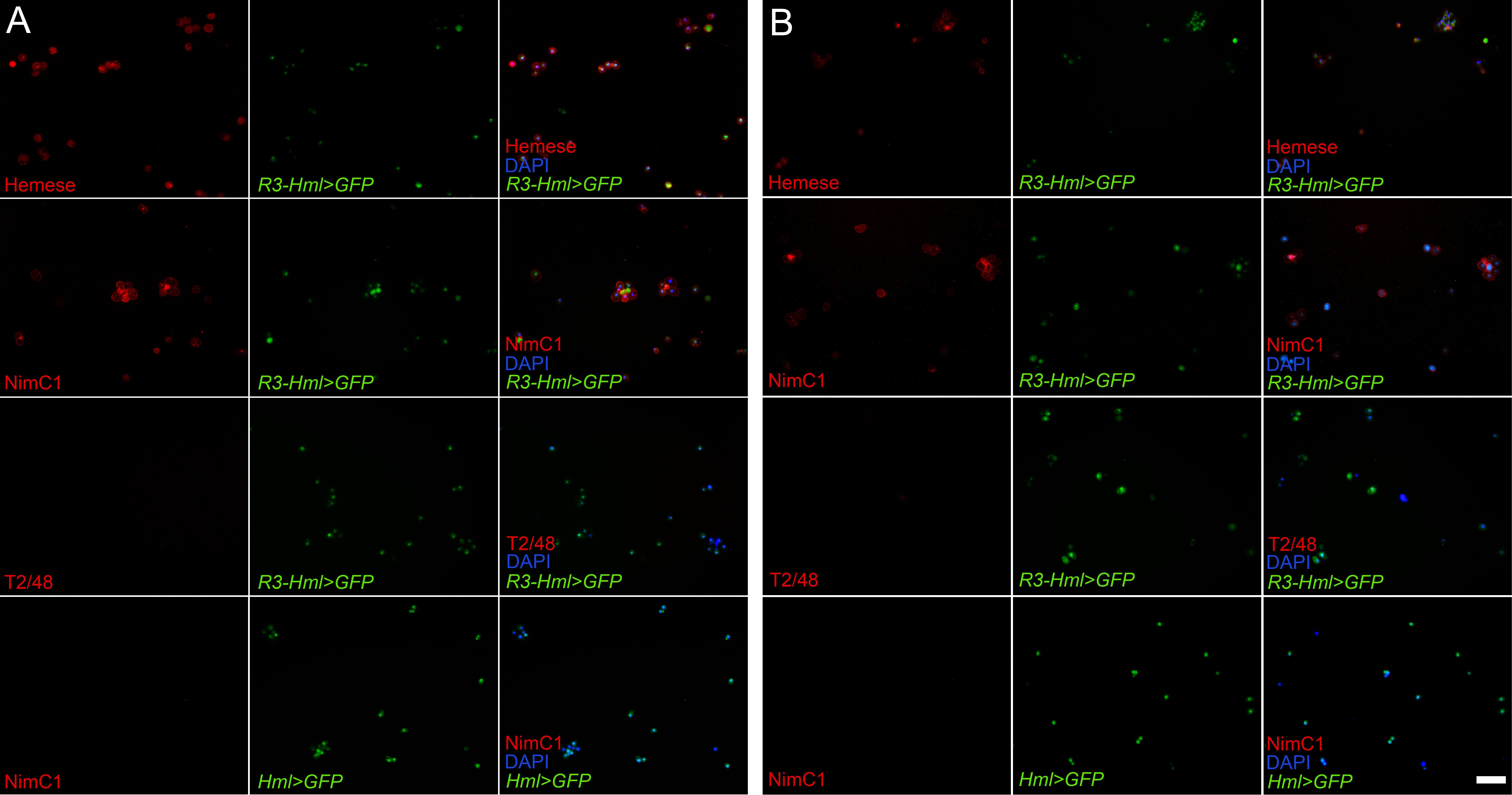

Supplement: Figure S1 — Immunostaining of circulating hemocytes with the sequential indirect immunofluorescent method (A), and the mixture of antibodies (B). The staining is shown in red, and the hemocytes are marked by their GFP expression (green), and DAPI nuclear staining (blue). The scale bar indicates 50 µm. (TIF) [file pone.0098191.s001.tif]
